# Impact of intracellular glyceraldehyde-derived advanced glycation end-products on human hepatocyte cell death

**DOI:** 10.1038/s41598-017-14711-3

**Published:** 2017-10-27

**Authors:** Akiko Sakasai-Sakai, Takanobu Takata, Jun-ichi Takino, Masayoshi Takeuchi

**Affiliations:** 1 Department of Advanced Medicine, Medical Research Institute, Kanazawa Medical University, Uchinada-machi, Ishikawa, 920-0293 Japan; 2 Department of Biochemistry, Faculty of Pharmaceutical Sciences, Hiroshima International University, Kure, Hiroshima, 737-0112 Japan

## Abstract

Hepatocyte cell death is a key feature of nonalcoholic steatohepatitis (NASH); however, the pathogenesis of NASH currently remains unclear. We aimed to investigate the effects of intracellular glyceraldehyde (GA)-derived advanced glycation end-products (GA-AGEs) on human hepatocyte cell death. The accumulation of intracellular GA-AGEs has been associated with the induction of DNA damage and hepatocyte necrotic cell death. Among intracellular GA-AGEs, caspase-3 has been identified as a GA-AGE-modified protein with abrogated protein function. Furthermore, the activation of caspase-3 and induction of hepatocyte apoptosis by camptothecin, a DNA-damaging agent, was suppressed by a treatment with GA. These results suggest the inhibitory effects of GA-AGE-modified caspase-3 on the induction of DNA-damage-induced apoptosis, which is associated with hepatocyte necrosis. Therefore, the suppression of necrosis, the inflammatory form of cell death, by the accumulation of GA-AGEs and GA-AGE-modified caspase-3 may represent a novel therapeutic target for the pathogenesis of NASH.

## Introduction

Nonalcoholic fatty liver disease (NAFLD) is currently the most common feature of chronic liver disease. The spectrum of NAFLD ranges from simple steatosis, steatohepatitis, fibrosis, and cirrhosis. Nonalcoholic steatohepatitis (NASH) is a severe form of NAFLD, and is characterized by hepatocellular lipid accumulation in addition to inflammation and fibrosis^1^. Since the suppression of inappropriate cell death associated with the pathogenesis of NASH may be a therapeutic target, the mechanisms responsible for cell death in NASH have been extensively examined. Hepatocyte apoptosis is a common feature of NASH. Apoptosis is a highly-regulated process of cell death that activates caspase family members including caspase-3, an effector of apoptosis, which is one of the prominent biochemical events that occur during apoptosis. Activated caspase-3 leads to the cleavage of poly(ADP-ribose) polymerase (PARP) for the manifestation of apoptosis. In addition to the large number of studies that have investigated the relationship between apoptosis and the progression of NASH, necrosis and necro-inflammation have also been histologically identified in NASH^2,3^. Apoptosis and necrosis are both involved in the pathogenesis of NASH and NASH-induced liver fibrosis; however, the factors responsible for and mechanisms underlying NASH-related cell death have not yet been elucidated in detail^4^.

NASH has been associated with metabolic syndrome, and a hyperglycemic condition is one of the risk factors for this disease^5,6^. In the hyperglycemic state, advanced glycation end-products (AGEs) are generated through a non-enzymatic glycation reaction (referred to as the Maillard reaction) between the ketone or aldehyde groups of the sugars and amino groups of proteins. AGEs exist in various forms depending on the sugar to be reacted. Glyceraldehyde (GA) is a metabolic intermediate of glucose and fructose, and GA-derived AGEs (GA-AGEs) are associated with NASH, infertility, cancer, dementia, schizophrenia, and cardiovascular disease^7–18^. Thus, GA-AGEs have been implicated in many diseases in various organs. However, GA-AGEs are expected to mainly accumulate in hepatocytes because fructose metabolism mostly occurs in the liver. The accumulation of GA-AGEs was previously reported in the liver tissues of patients with NASH, but less in simple steatosis^7^. Furthermore, we showed that serum levels of GA-AGEs were significantly higher in NASH patients than in those with simple steatosis or healthy controls^7^. GA-AGEs accumulate in NASH patients, and also exhibit strong cytotoxicity when they gather in cells. We previously reported that the treatment of the human hepatocellular carcinoma (HCC) cell line Hep3B with GA or high doses of fructose resulted in the accumulation of GA-AGEs in these cells, and also identified heat shock cognate 70 (Hsc70) or heterogeneous nuclear ribonucleoprotein M (hnRNPM) as a GA-AGE-modified protein^19,20^. GA-AGE-modified Hsc70 lost its chaperone activity and correlated with hepatocyte cell death. In addition to the accumulation of GA-AGEs, the mRNA of the inflammatory marker C-reactive protein (CRP) was significantly increased in Hep3B cells by a treatment with GA^19^. These findings suggest that the accumulation of GA-AGE-modified intracellular proteins causes cellular dysfunction and induces inflammatory responses. However, the cell death type and mechanisms induced by the accumulation of GA-AGEs in hepatocytes, which we proposed as one of the causes of NASH, currently remain unclear.

In the present study, we investigated the cell death type and mechanisms induced by the accumulation of intracellular GA-AGEs in human hepatocytes, and identified GA-AGE-modified proteins. The accumulation of GA-AGEs in the human HCC cell line, HepG2, induced DNA damage and necrotic cell death. This necrosis appeared to correlate with the anti-apoptotic effects induced by GA-AGE modifications to caspase-3. Our results provide novel insights into cell death associated with NASH, which has potential as a therapeutic anti-inflammation target for the treatment of NASH.

## Results

### Accumulation of intracellular GA-AGEs induces cytotoxicity in human hepatocytes

**Figure 1:**
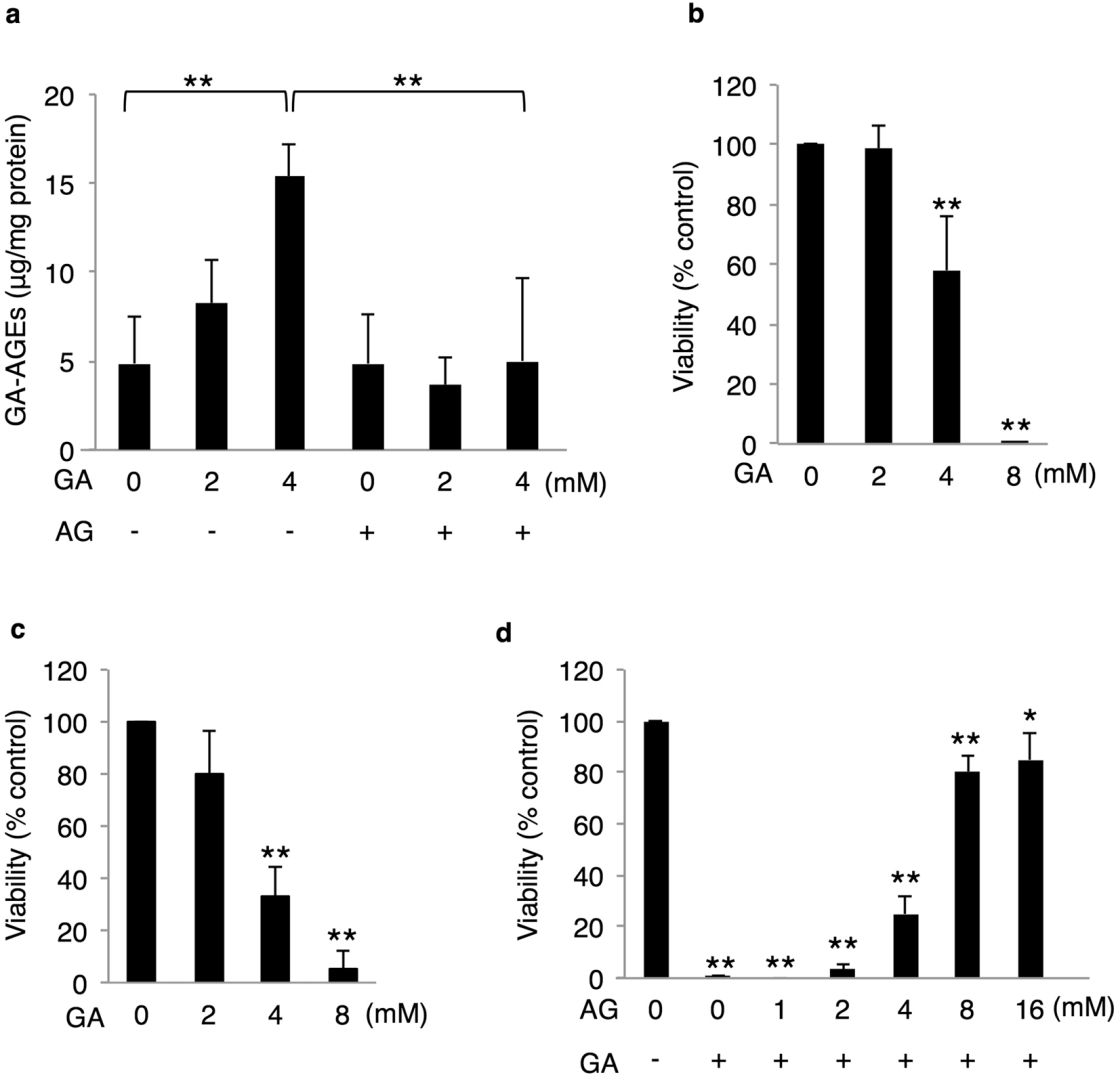
Accumulation of intracellular GA-AGEs induced human hepatocyte cell death. (**a**) Slot blotting was performed to measure intracellular GA-AGEs (n = 4). Cell extracts were prepared from HepG2 cells pretreated with 0 or 16 mM AG for 2 h followed by 0, 2, and 4 mM GA for 24 h. (**b**–**d**) Cell viability was measured by the CellTiter-Glo luminescent cell viability assay (n = 3). HepG2 cells (**b**) or primary hepatocytes (**c**) were treated with various concentrations of GA. (**d**) HepG2 cells were treated with 0, 1, 2, 4, 8, and 16 mM AG for 2 h, followed by 0 or 8 mM GA for 24 h. Results are means ± S.D. *p < 0.05 and **p < 0.01 based on a one-way ANOVA followed by Tukey’s test (**a**) or Dunnett’s test (**b**–**d**).

GA-AGEs are expected to mainly accumulate in hepatocytes because fructose metabolism mostly occurs in the liver. In order to focus on the effects of intracellular GA-AGEs, we used HepG2 cells, which are not affected by extracellular GA-AGEs^21,22^. In an attempt to clarify whether intracellular GA-AGEs accumulate in hepatocytes following a treatment with GA, HepG2 cells were treated with GA and cell extracts were analyzed by slot blotting with an anti-GA-AGE antibody to measure the accumulation of intracellular GA-AGEs. The accumulation of intracellular GA-AGEs was increased by the GA treatment and suppressed by a pre-incubation with aminoguanidine (AG), an inhibitor of AGE formation (Fig. 1a). We then assessed the cytotoxicity of intracellular GA-AGE accumulation. The GA treatment induced cell death in a GA dose-dependent manner in HepG2 cells (Fig. 1b). We confirmed this cytotoxicity in human primary hepatocytes as well as HepG2 cells (Fig. 1c). GA-induced cytotoxicity in HepG2 cells was rescued by a pre-incubation with AG in a dose-dependent manner (Fig. 1d). These results suggest that the accumulation of GA-AGEs induces cell death in human hepatocytes.

### The GA treatment induces DNA damage in HepG2 cells

**Figure 2:**
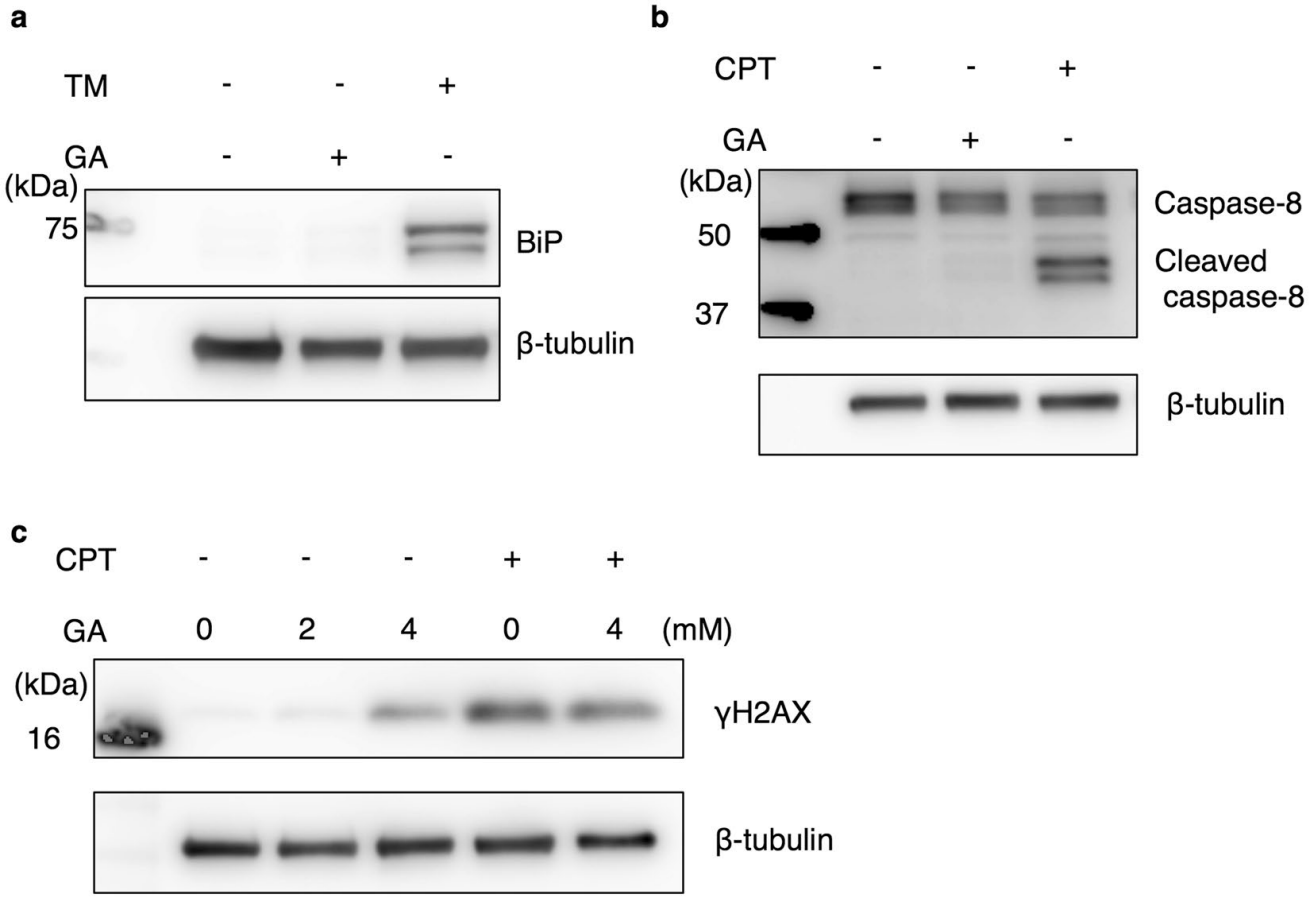
DNA damage in HepG2 cells treated with GA. Western blotting (WB) was performed to analyze the effects of the GA treatment on cellular stress responses in HepG2 cells. (**a,b**) Cell extracts were prepared from HepG2 cells treated with vehicle (DMSO) or 4 mM GA and incubated for 24 h. In analyses of the positive control of stress responses, cell extracts were prepared from HepG2 cells treated with 10 µg/ml tunicamycin (TM) (**a**) or 10 µM CPT (**b,c**) for 22 h. The WB analysis was conducted using an anti-BiP antibody (**a**), anti-caspase-8 antibody (**b**), and anti-γH2AX antibody (**c**). β-tubulin was used as a loading control. Full-length blots are presented in Supplementary Fig. S2.

Our results and previous findings demonstrated that GA induces cell death^19^. The underlying mechanisms may involve various functional disorders in intracellular components and composite stress responses, but currently remain controversial. In order to clarify cellular stress induced by the GA treatment, we initially examined whether the endoplasmic reticulum (ER) stress response or death receptor signaling pathway is activated by the GA treatment because both were previously reported to be important for hepatocyte disorders in the pathology of NASH^23,24^. The expression of the ER stress marker protein, BiP, was strongly up-regulated by tunicamycin-induced ER stress (Fig. 2a)^25^. Furthermore, the cleavage of caspase-8, a marker for the activation of the death receptor pathway, was promoted by the camptothecin (CPT) treatment, as previously reported (Fig. 2b)^26^. However, we did not observe both events in HepG2 cells treated with GA (Fig. 2a,b). On the other hand, AGE modifications may also occur in various proteins and DNA^27,28^. We measured the phosphorylated histone, H2AX (γH2AX) as a marker of DNA damage. Increases in γH2AX were induced by the genotoxic agent CPT, as previously reported^29^. The induction of γH2AX was also observed in HepG2 cells treated with GA (Fig. 2c). These results suggested that DNA damage, but not induction of ER stress or activation of cell death receptor pathways, induced by in HepG2 cells treated with GA.

### Accumulation of intracellular GA-AGEs induces necrotic cell death in HepG2 cells

**Figure 3:**
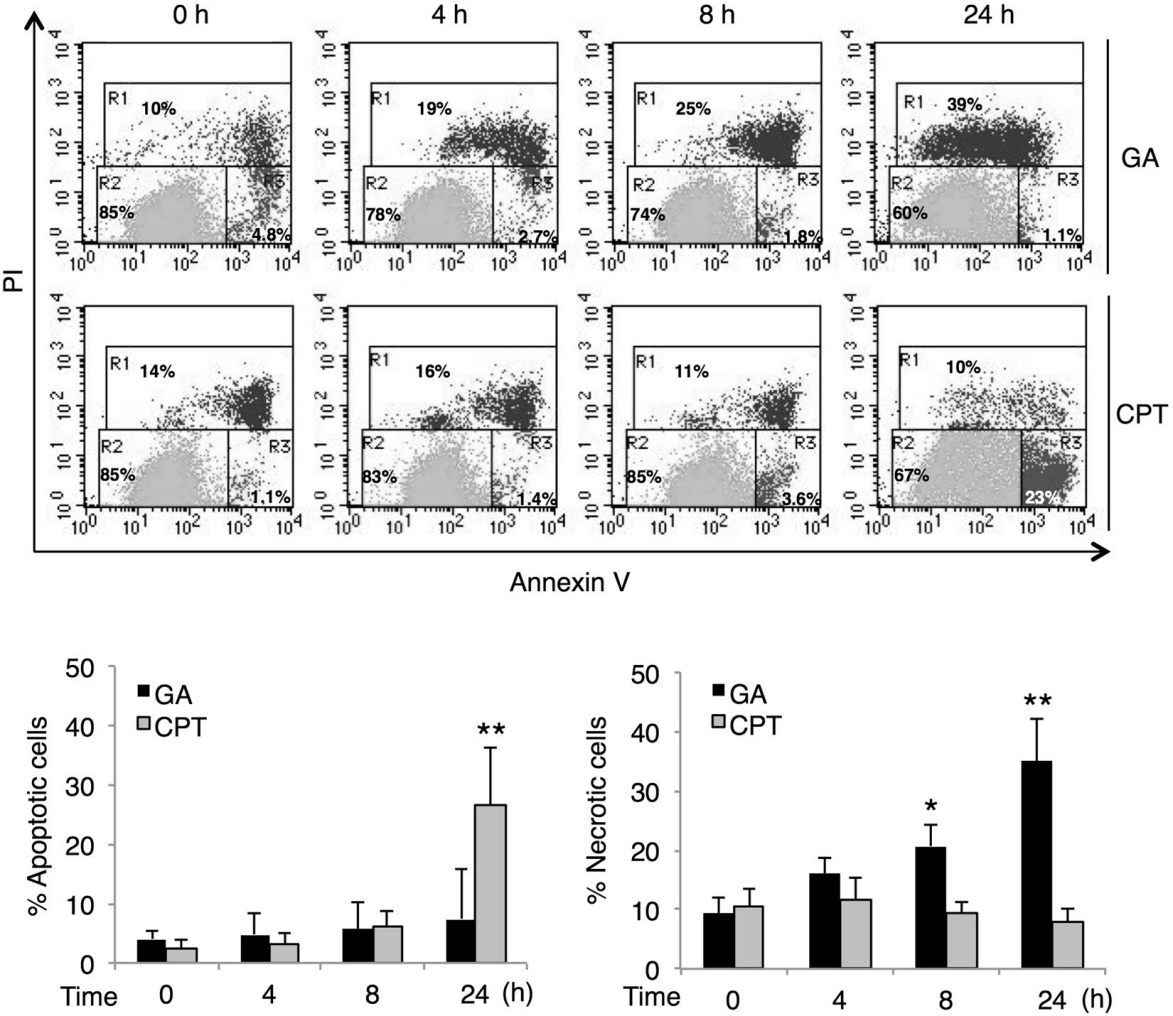
Necrotic cell death is induced in HepG2 cells treated with GA. The effects of the GA treatment on cell death were analyzed using PI/annexin V staining and flow cytometry. HepG2 cells were treated with 0 or 4 mM GA, and 10 µM CPT or vehicle (DMSO), and incubated for the desired time prior to being stained. The X-axis and Y-axis represent the fluorescence intensity of annexin V and propidium iodide (PI), respectively. The region R was defined as R1 = necrotic cells, R2 = viable cells, and R3 = apoptotic cells (upper panel). The percentages of apoptotic and necrotic cells were presented as means ± S.D. (lower panel). *p < 0.05 and **p < 0.01 (n = 3) based on a one-way ANOVA followed by Dunnett’s test.

In order to elucidate the type of cell death induced by the GA treatment, HepG2 cells were labeled with FITC-conjugated annexin V and propidium iodide (PI) after the GA treatment. Apoptotic cells are detected by annexin V staining because phosphatidylserine is translocated to the outer membrane; however, PI is excluded from these cells (annexin V-positive/PI-negative). The plasma membrane in necrotic cells is compromised and DNA is stained by PI (PI-positive). These cell death types were defined on a flow cytometry chart as R1 = necrotic cells, R2 = viable cells, and R3 = apoptotic cells (Fig. 3). HepG2 cells were also incubated with CPT as a positive control for apoptosis. The percentage of apoptotic cells after 24 h was higher with the CPT treatment than with the vehicle treatment, whereas that of necrotic cells was not increased. On the other hand, the percentage of GA-treated HepG2 cells increased in the necrotic fraction, but not in the apoptotic fraction in a time-dependent manner. Apoptotic cells progressively leak cellular components when phagocytes do not function and are detected as necrotic cells (termed secondary necrosis)^30^. In the case of the GA treatment, increases in necrosis did not follow apoptosis, suggesting that the enhancement in HepG2 cell death by the GA treatment was due to necrosis, not apoptosis or secondary necrosis. Furthermore, necrotic cell death was observed in GA-treated human primary hepatocytes (Supplementary Fig. S1).

### Caspase-3 in hepatocytes is modified by GA-AGEs and multimerized by the GA treatment

**Figure 4:**
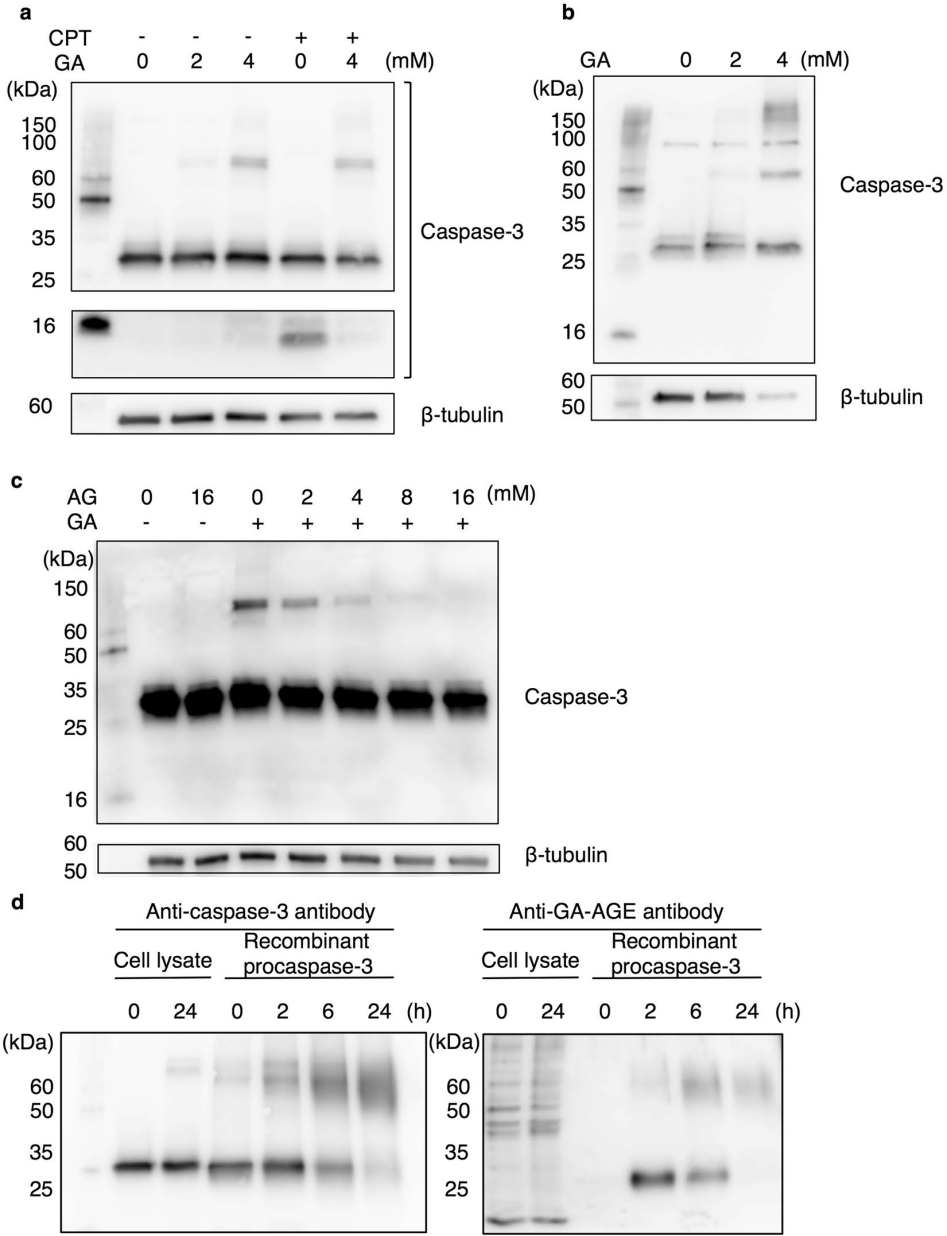
Hepatocyte caspase-3 is modified by GA-AGEs following a treatment with GA. (**a**,**b**) Western blotting (WB) was performed to analyze the effects of the GA treatment on intracellular caspase-3. (**a**) Cell extracts were prepared from HepG2 cells treated with 0, 2, or 4 mM GA for 2 h followed by 10 µM CPT or vehicle (DMSO) for 22 h. (**b**) Cell extracts were prepared from primary hepatocytes after a treatment with 0, 2, or 4 mM GA for 24 h. Full-length blots are presented in Supplementary Figs. S3 and S4, respectively. (**c**) HepG2 cells were pretreated with 0, 2, 4, 8, and 16 mM AG for 2 h followed by 0 or 4 mM GA. Cell extracts were analyzed by WB. Full-length blots of β-tubulin are presented in Supplementary Fig. S5. (**d**) GA-treated cell extracts and recombinant procaspase-3 were analyzed by WB. Cell extracts were prepared from HepG2 cells after a treatment with 0 or 4 mM GA for 24 h. A total of 0.75 µM recombinant procaspase-3 was incubated with 4 mM GA for 0, 2, 6, and 24 h. Full-length blots are presented in Supplementary Fig. S6. The WB analysis was conducted using an anti-caspase-3 antibody (**a–d**) and anti-GA-AGE antibody (**d**). β-tubulin was used as a loading control.

In order to further analyze the relationship between GA-induced DNA damage and necrosis, we examined the behavior of caspase-3. Caspase-3 exists as an inactive dimer called procaspase-3 under non-apoptotic conditions, but is cleaved when the apoptotic pathway is activated^31^. We found slower migrating caspase-3 bands in GA-treated HepG2 cells (Fig. 4a) as well as in primary human hepatocytes by Western blotting (WB) (Fig. 4b). These slower migrating bands in GA-treated HepG2 cell extracts disappeared in a dose-dependent manner with an AG pretreatment (Fig. 4c). The slower migrating bands were suggested to be caspase-3 multimers because glycated proteins are known to form crosslinking structures on adjacent proteins or within the domains of a protein^32^. In order to clarify whether these slower migrating bands appeared due to GA-AGE modifications to caspase-3, we attempted to detect GA-AGE-modified caspase-3 bands with an anti-GA-AGE antibody in GA-treated HepG2 cell extracts. However, since a large number of bands were observed in GA-untreated and -treated cell extracts with the anti-GA-AGE antibody, we were unable to identify GA-AGE-modified caspase-3 bands (Fig. 4d, lanes 1 and 2 in the right panel). We then used a recombinant procaspase-3 protein and focused on its modification. The recombinant procaspase-3 protein was treated with GA *in vitro*, slower migrating caspase-3 bands were detected with the anti-caspase-3 antibody, and the intensity of the bands increased in a time-dependent manner (Fig. 4d, left panel). Furthermore, the analysis with the antibody against GA-AGEs revealed that GA-treated procaspase-3 was detected as a monomer and slower migrating band, whereas vehicle-treated procaspase-3 was not (Fig. 4d, right panel). The monomer caspase-3 detected with the anti-GA-AGE antibody gradually decreased, whereas the slower migrating caspase-3 bands increased in a GA treatment time-dependent manner. Slower migrating recombinant caspase-3 is considered to be a GA-AGE-modified caspase-3 multimer and was of a similar size to the slower migrating cellular bands. These results suggested that hepatocyte caspase-3 was modified by GA-AGEs with the GA treatment, and this modification induced the multimerization of caspase-3.

### GA-AGE modifications to caspase-3 abrogate its activity for the execution of apoptosis

The caspase-3 protein is cleaved and exerts its enzymatic effects during apoptosis. Thus, we attempted to elucidate whether GA-AGE-modified caspase-3 is still functional for apoptosis. We initially investigated the effects of the GA treatment on caspase-3 cleavage using WB. CPT was employed as a positive control for apoptosis, and caspase-3 was detected as cleaved bands in cells treated with CPT alone, but only slightly in the GA-treated HepG2 cell extract (Fig. 4a). However, the CPT-induced cleavage of caspase-3 was suppressed by a combination with the GA pretreatment (Fig. 4a).

**Figure 5:**
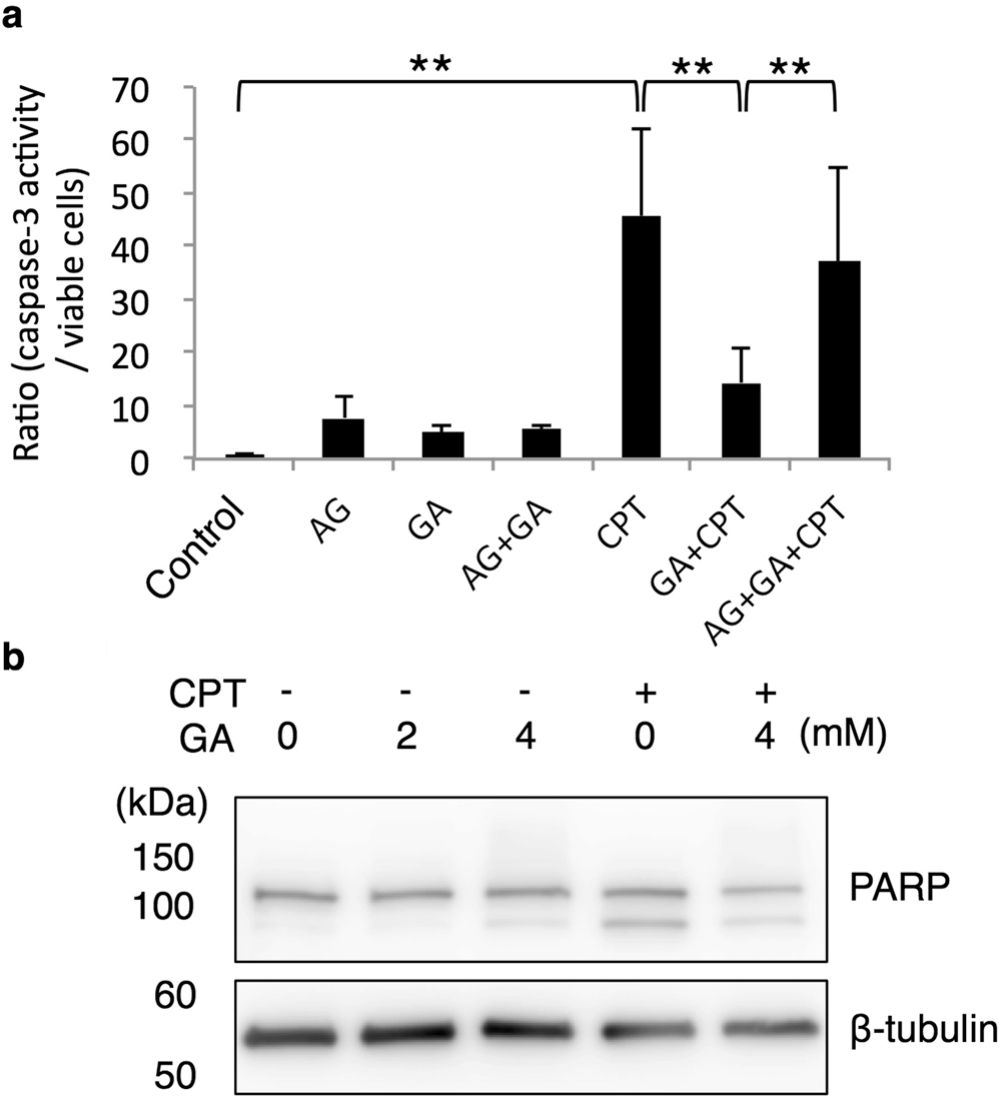
GA-AGE-modified caspase-3 exhibits abrogated enzyme activity. (**a**) The cell viability and caspase-3 activity of GA-treated HepG2 cells were measured by the CellTiter-Glo luminescent cell viability assay and Caspase-Glo 3/7 assay, respectively (n = 4). HepG2 cells were treated with 0 or 16 mM AG for 2 h followed by 0 or 4 mM GA for 2 h, and then incubated with 10 µM CPT or vehicle (DMSO) for 22 h. The ratio represented caspase-3 activity calculated by dividing the value from the Caspase-Glo 3/7 assay by that from the CellTiter-Glo luminescent cell viability assay. Results are means ± S.D. *p < 0.05 and **p < 0.01 based on a one-way ANOVA followed by Tukey’s test. (**b**) WB was performed to analyze the effects of the GA treatment on PARP cleavage. Cell extracts were prepared from HepG2 cells treated with 0, 2, or 4 mM GA for 2 h followed by 10 µM CPT or vehicle (DMSO) for 22 h. Full-length blots are presented in Supplementary Fig. S7. The WB analysis was conducted using an anti-PARP antibody. β-tubulin was used as a loading control.

In order to clarify whether GA-AGE-modified caspase-3 exhibits enzymatic activity, it was directly measured in a cell-based assay (Fig. 5a). The enzyme activity of caspase-3 was markedly stronger with the CPT treatment than with the control treatment, whereas CPT-induced caspase-3 activation was markedly suppressed in combination with the GA pretreatment in HepG2 cells. Furthermore, the suppressive effects of GA on the activation of caspase-3 were restored by the additional treatment with AG. WB revealed that the CPT-induced cleavage of PARP, a well-known substrate of caspase-3, was also significantly cleaved by the CPT treatment, but only slightly in GA-treated HepG2 cells. The GA pretreatment suppressed CPT-induced PARP cleavage as well as caspase-3 modifications (Fig. 5b). In order to exclude the possibility that the GA treatment decreases the amount of DNA damage induced by CPT in HepG2 cells, we assessed γH2AX (Fig. 2c). The treatment with GA prior to CPT did not decrease γH2AX. This result indicated that the GA pretreatment does not disturb CPT-induced DNA damage signaling. Collectively, these results suggested that GA-AGE modifications disturbed the activation of caspase-3 for the execution of apoptosis.

### The GA treatment suppresses CPT-inducing apoptosis and causes necrosis in HepG2 cells

**Figure 6:**
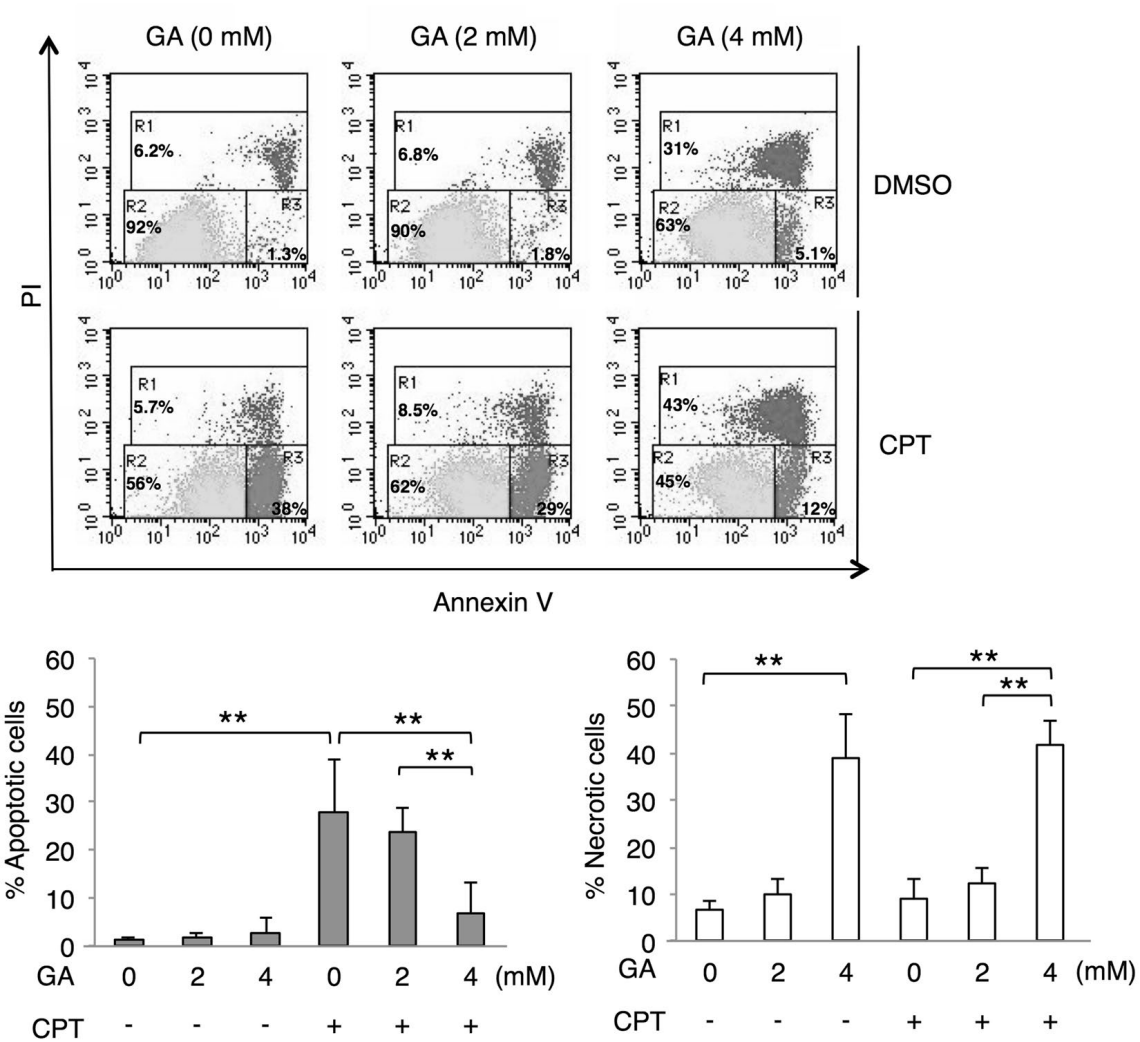
Apoptotic cell death induced by CPT is suppressed by a treatment with GA. HepG2 cells were treated with 0, 2, and 4 mM GA for 2 h followed by 10 µM CPT or vehicle (DMSO) and incubated for 22 h prior to being stained. The X-axis and Y-axis represent the fluorescence intensities of annexin V and PI, respectively. The region R was indicated as R1 = necrotic cells, R2 = viable cells, or R3 = apoptotic cells (upper panel). The percentages of apoptotic and necrotic cells were presented as means ± S.D. (lower panel). *p < 0.05 and **p < 0.01 (n = 5) based on a one-way ANOVA followed by Tukey’s test.

The suppression of caspase-3 activation and PARP cleavage by the GA treatment allowed us to analyze the effects of GA on the execution of apoptosis in more detail. We also investigated the effects of GA on HepG2 cell death induced by CPT (Fig. 6). Necrosis, but not apoptosis was enhanced more in GA-treated HepG2 cells than in non-treated cells, similar to the results shown in Fig. 3. The CPT treatment efficiently induced apoptosis in HepG2 cells, whereas the pretreatment with GA suppressed CPT-induced apoptosis and led to necrosis. These results suggested that the suppressive effects of the GA treatment on CPT-induced apoptosis correlated with the loss of CPT-induced caspase-3 activity for the execution of apoptosis by GA-AGE modifications.

## Discussion

The “multiple parallel hits” hypothesis has been used to explain the progression of NAFLD to NASH^33^. “Multiple” indicates insulin resistance, nutritional factors, host genetics, and gut microflora. These hits occur in parallel and ultimately result in liver inflammation^33,34^. In addition, AGEs are produced at a high frequency in a hyperglycemic state and are considered to be associated with the pathogenesis of NASH^35^. Among AGEs, GA-AGEs influence hepatocytes and hepatic stellate cells (HSCs) intra- and extracellularly^19–22,36,37^. We hypothesized that GA-AGEs are one of the “multiple parallel hits”. However, the cell death mechanisms induced by the accumulation of GA-AGEs in hepatocytes currently remain unclear. Moreover, *in vivo* and intracellular concentrations of GA remain uncertain. In order to investigate the impact of intracellular GA-AGE accumulation, we extracellularly added GA in the millimolar order. Previous studies reported that fructose is a major cause of GA and fructose-fed mice may exhibit a NASH-like pathology^35,38^. Even at the cellular level, we confirmed the accumulation of GA-AGE-modified proteins in hepatocytes incubated with a high fructose concentration for 5 days, and the accumulation of these proteins was also observed when these cells were incubated with 4 mM GA for 6 h^20^. Therefore, the GA treatment in the millimolar order is considered to be the optimum concentration for short-term experiments using cultured cells.

In the present study, we investigated the types of and mechanisms underlying human hepatocyte cell death induced by the accumulation of intracellular GA-AGEs and identified GA-AGE-modified proteins. Regarding the cell death type, apoptotic cell death in hepatocytes is a common feature of NASH^4^. However, we demonstrated that the accumulation of GA-AGEs induced necrosis instead of apoptotic cell death, contrary to our expectation (Figs 3 and 6). Previous studies reported that in addition to apoptosis, necrosis and necro-inflammation have been observed histologically in NASH, and regulated necrosis was proposed as a new model of cell death in NASH^3,39^. This finding prompted us to hypothesize that necrotic cell death induced by the accumulation of intracellular GA-AGEs is involved in the pathogenesis of NASH.

In addition to the type of cell death, the involvement of caspase-3 in the pathogenesis of NASH currently remains unclear. We identified hepatocyte caspase-3 as a GA-AGE-modified protein (Fig. 4). Previous studies reported that glycated proteins form crosslinking structures, and heterogeneous sizes and charge properties exist due to the AGE biosynthesis of proteins, which are detected as smear bands^32,40,41^. We identified the slower migrating bands of caspase-3 as a smear band following the GA treatment *in vitro*, and these bands were of similar sizes to the caspase-3 bands detected in GA-treated HepG2 cells (Fig. 4). Consequently, these results suggested that caspase-3 in GA-treated cells is multimerized by GA-AGE modifications.

**Figure 7:**
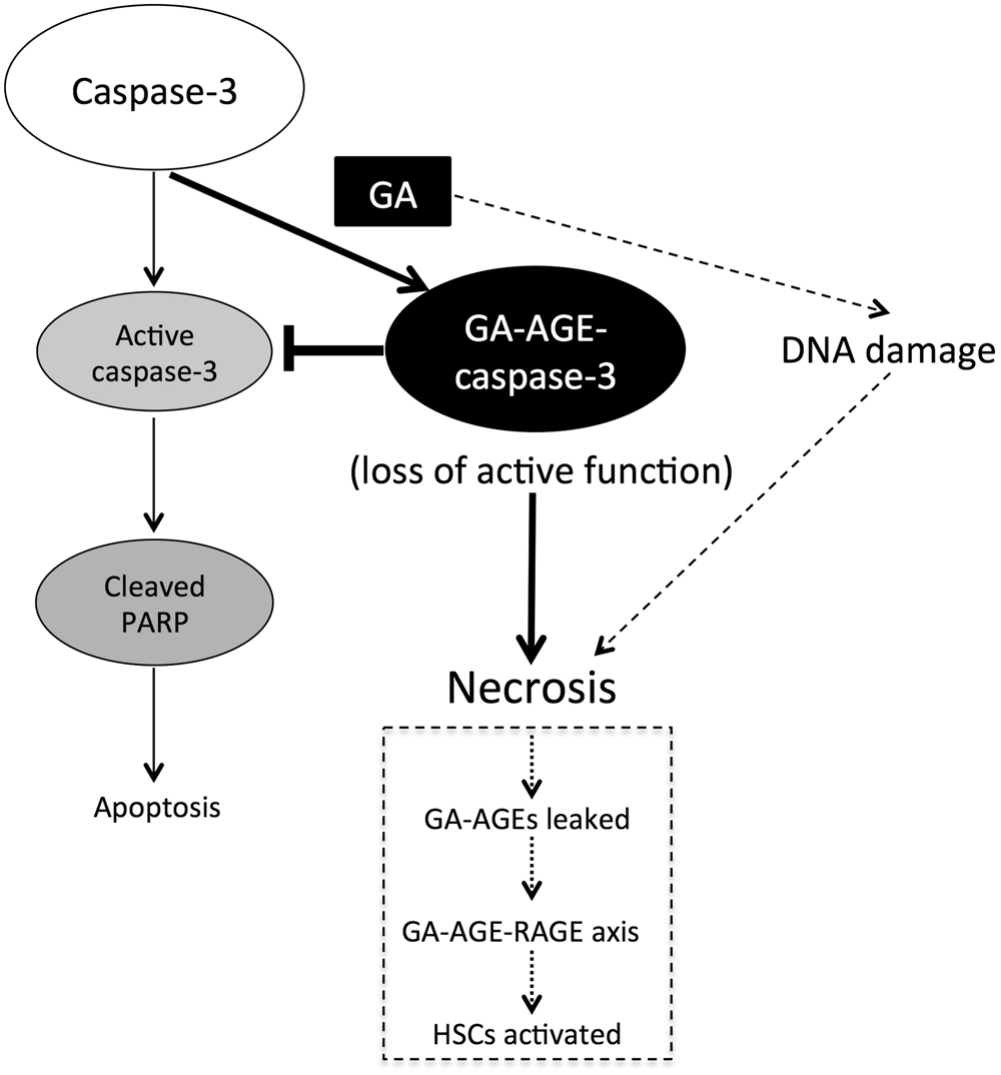
A proposed mechanism depicting the role of GA in HepG2 cell death. GA induces DNA damage and GA-AGE-caspase-3. This cellular damage is associated with the suppression of active caspase-3, followed by cleaved PARP, and apoptotic cell death. GA: glyceraldehyde, PARP: poly(ADP-ribose) polymerase, RAGE: receptor for AGEs, HSCs: hepatic stellate cells.

Caspase-3 exists as an inactive form of procaspase-3, but is converted to the cleaved form and activated under apoptotic conditions. Activated caspase-3 cleaved PARP for the manifestation of apoptosis. These apoptotic cascades are induced by several cellular stresses, including DNA damage. The present results suggest that DNA damage in HepG2 cells was caused by the GA treatment (Fig. 2). GA has the potential to damage DNA directly or indirectly because AGE modifications may occur in DNA and various proteins^27,28^. In addition to the induction of γH2AX as a DNA damage response, γH2AX has been observed in the late stage of apoptosis, in which DNA fragmentation starts. However, apoptosis was not significantly induced by the GA treatment (Figs 3 and 6), indicating that this γH2AX signal is derived from DNA damage. Consistent with GA-induced DNA damage, γH2AX was previously detected in the livers of mice fed a high-fat diet^42^. In this study, we used CPT as a control for apoptosis that causes DNA double-strand breaks mediated by DNA topoisomerase 1 and eventually induces cell death in various cancer cells including hepatocytes^43,44^. CPT significantly induced DNA damage as well as the activation of caspase-3 and PARP cleavage in HepG2 cells, whereas these events were suppressed by the GA pretreatment (Figs 4 and 5). The GA pretreatment also suppressed CPT-induced apoptotic cell death and caused necrosis (Fig. 6). These results indicate that the activation of caspase-3 in the apoptotic cascade induced by DNA damage is suppressed by GA-AGE-modified caspase-3, leading to necrotic cell death (Fig. 7). On the other hand, the intensity of the multimerized caspase-3 band induced by the GA treatment was less than that of the monomer band. The recombinant caspase-3 protein was GA-AGE-modified to the monomer form and gradually multimerized. These results prompted us to hypothesize that a large proportion of intracellular caspase-3 is modified by GA-AGEs without becoming a multimer. Further analyses are needed on the mechanisms responsible for converting GA-AGE-modified caspase-3 into a multimer in HepG2 cells.

Previous studies reported that hepatocyte caspase-3 activity is involved in the pathogenesis of NASH; however, the role of active caspase-3 during the course of NASH may be altered^45–48^. In the early stage of NASH, caspase-3 is not activated in patients with mild NASH, whereas caspase-3 activity has been detected in those with severe NASH^45^. Moreover, caspase-3-knockout mice and wild-type mice fed a methionine choline-deficient (MCD) diet showed similar liver injury^46^. In the advanced stage of NASH, in which hepatic fibrosis occurs, the inhibition of caspase suppressed liver fibrosis^47,48^. These findings on caspase-3 prompted us to propose that apoptosis accompanied by the activation of caspase-3 is not the main cause of the “onset” of NASH, whereas necrosis induced inflammatory responses related to pathogenesis in the early stage of NASH. Necrotic hepatocyte cell death induced by the accumulation of GA-AGEs is presumed to release cellular GA-AGEs and inflammatory substances, including CRP. We previously demonstrated that CRP mRNA levels were significantly increased in hepatocytes by a GA treatment^19^. Regarding extracellular GA-AGEs, apoptotic cell death is induced via the receptor for AGE (RAGE) interaction, for example, in β cells of the pancreas^49^. Extracellular AGEs are involved not only in apoptosis, but also in the activation of HSCs, which participate in hepatic fibrosis^36,37,50,51^. Therefore, we speculate that the accumulation of GA-AGEs causes hepatic necrosis in the early phase of NASH, the leakage of GA-AGEs from necrotic cells induces apoptosis via RAGE in caspase-3 intact cells, and HSCs are activated in the progressive stages of NASH. Further studies are needed in order to clarify whether the glycation of caspase-3 is responsible for inducing necrotic cell death; however, our results provide a novel insight into the cell death mechanisms involved in the pathogenesis of NASH.

## Materials and Methods

### Reagents and Antibodies

Glyceraldehyde (GA) was purchased from Nacalai Tesque. Aminoguanidine (AG) was from Wako Chemical Industries, Ltd.. Camptothecin (CPT) was obtained from Sigma. The following antibodies were used in the present study: anti-caspase-3, anti-PARP (Cell Signaling Technology); anti-β-tubulin (Wako); and an anti-γH2AX antibody (Millipore). An anti-GA-AGE antibody was prepared and purified as described previously^41^. A horseradish peroxidase (HRP)-linked anti-rabbit IgG antibody and HRP-linked anti-mouse IgG antibody were obtained from Cell Signaling Technology.

### Cell Culture

The human HCC cell line, HepG2 (ECACC No. 85011430) was purchased from ECACC and maintained in low glucose Dulbecco’s modified Eagle’s medium (DMEM; Sigma) supplemented with 10% fetal bovine serum (FBS; Sigma), 100 U/ml penicillin, and 100 µg/ml streptomycin (Wako). Primary cultured cells of hepatocytes from a human adult liver were purchased from Kurabo and maintained in KLC seeding medium (Kurabo). These cells were kept at 37 °C in a humidified incubator with 5% CO_2_. Cells were plated at a density of 3.0 × 10^4^ cells/cm^2^ for HepG2 cells or 4.0 × 10^4^ cells/cm^2^ for human primary hepatocytes. Treatments with reagents including GA were performed in 2% FBS/DMEM in order to prevent the formation of extracellular AGEs.

### Slot blotting

Slot blotting was performed to detect the total amount of GA-AGEs in floating and adherent hepatocyte cell extracts treated with the desired drug treatments. This analysis was performed as previously described with some modifications^52^. Briefly, floating and adherent cells were washed with PBS (−) and lysed in buffer [4% CHAPS, 30 mM Tris, 2 M Thiourea, 7 M Urea, and protease inhibitor cocktail (complete Mini; Roche)]. Cell extracts were then incubated on ice for 5 min, centrifuged at 10,000 × *g* at 4 °C for 10 min, and the supernatant was collected as the cell extract. Protein concentrations were measured using the XL-Bradford assay kit (APRO Science). GA-AGE-modified bovine serum albumin (BSA) was prepared as described in our previous study^41^, and used for a calibration curve to measure the amount of GA-AGEs in cell extracts. In the detection of GA-AGEs, equal amounts of cell extracts were loaded onto polyvinylidene fluoride (PVDF) membranes (0.45 µm; Millipore) fixed in a slot blot apparatus (Bio-Rad). Samples were then blotted under vacuum conditions. Membranes were blocked at room temperature for 1 h using 5% skimmed milk in PBS containing 0.05% Tween 20 (PBS-T) followed by washing twice with PBS-T. Membranes were incubated with an anti-GA-AGE antibody at a dilution of 1:1,000 at 4 °C overnight. Membranes were then washed twice with PBS-T and incubated with the HRP-linked anti-rabbit IgG antibody at a dilution of 1:2,000. After being washed two more times with PBS-T, immunoreactive proteins were detected with Chemi-Lumi One L (Nacalai Tesque) using a luminescent image analyzer (LAS-4000; GE-Healthcare).

### Cell viability and caspase-3 activity assays

The CellTiter-Glo luminescent cell viability assay and Caspase-Glo 3/7 assay were performed on each sample according to the manufacturer’s instructions (Promega). Briefly, cells were plated in triplicate onto white opaque 96-well plates. After an incubation for 24 h, cells were treated with the indicated reagent for the desired time period. Cells were then incubated for 10 min with CellTiter-Glo reagent or 2 h with Caspase-Glo reagent, and luminescence was measured using a 96-well plate reader (GloMax-96 microplate luminometer; Promega). Background luminescence was measured in medium without cells and subtracted from experimental values. Neither GA nor AG exerted suppressive effects on luciferase activity in this assay.

### Western blotting

Floating and adherent cells were washed with PBS (−) three times and lysed in sodium dodecyl sulfate (SDS) sample buffer (Bio-Rad) and 2-mercaptoethanol (Sigma-Aldrich), followed by heating at 95 °C for 5 min. Equal amounts of cell extracts were resolved by SDS polyacrylamide gel electrophoresis and transferred onto PVDF membranes. The membranes were blocked at room temperature for 1 h using 5% skimmed milk in PBS-T, followed by washing twice with PBS-T. The membranes were incubated with the anti-GA-AGE antibody (1:1,000), anti-caspase-3 antibody (1:3,000), anti-PARP antibody (1:1,000), anti-BiP antibody (1:1,000), anti-caspase-8 antibody (1:1,000), or anti-γH2AX antibody (1:1,000) at 4 °C overnight. Washing and an incubation with the secondary antibody were performed as described in the slot blotting section. Immunoreactive proteins were detected with Chemi-Lumi One Super (Nacalai Tesque) using a luminescent image analyzer (LAS-4000; GE-Healthcare). Equivalent sample loading was confirmed by stripping membranes with the Ten Minute Western Blot Re-Probe Kit (Funakoshi), followed by blotting with the anti-β-tubulin antibody (1:30,000).

### Annexin V-FITC flow cytometric analysis

The Dead Cell Apoptosis Kit with annexin V-FITC and PI for flow cytometry (Invitrogen) was used to detect viable, apoptotic, and necrotic cells. In brief, HepG2 cells were treated with various concentrations of GA for 24 h. In the case of CPT, HepG2 cells were treated with GA for 2 h followed by 10 µM CPT or a vehicle (dimethyl sulfoxide, DMSO) for 22 h. Floating and trypsinized cells were washed in ice-cold PBS with 0.5% BSA and stained using the Dead Cell Apoptosis Kit according to the manufacturer’s instructions. A fluorescent-activated cell analysis was performed using a FACSCalibur flow cytometer (Becton Dickinson), and 10,000 events were represented as dot plots.

### *In vitro* GA-AGE modification assay of caspase-3

A total of 0.75 µM recombinant human procaspase-3 (R&D Systems) was incubated with 4 mM GA in 20 mM phosphate buffer (pH 7.4) for the indicated period. These reaction mixtures were denatured by adding SDS sample buffer followed by heating at 95 °C for 5 min.

### Statistical analysis

We used a one-way ANOVA followed by Tukey’s test or Dunnett’s test for comparisons of intergroup differences using Stat Flex 6.0 software (Artech) and representative graphs have been prepared. Experiments were repeated at least three times and data are presented as the mean ± S.D. Significant differences are presented as *P*-values < 0.05 or < 0.01 in the figures and corresponding figure legends.

## Electronic supplementary material

Supplemental Figures
