# Supplementary material for: Impact of intracellular glyceraldehyde-derived advanced glycation end-products on human hepatocyte cell death
Source: Sci Rep. 2017 Oct 27;7:14282. doi: 10.1038/s41598-017-14711-3 (PMC5660208; doi:10.1038/s41598-017-14711-3)
Supplement: Supplementary file 1 — Supplemental Figures [file 41598_2017_14711_MOESM1_ESM.pdf]

Supplemental Information

Impact of intracellular glyceraldehyde-derived advanced glycation end-products on human hepatocyte cell death

Akiko Sakasai-Sakai, Takanobu Takata, Jun-ichi Takino, & Masayoshi Takeuchi

Materials and methods

Detection of Annexin V/PI staining cells under fluorescent microscope.

Cells grown on 12-mm collagenized coverslips in 24-well plates were washed with cold-PBS and then washed with 1×Annexin-binding buffer. These cells were stained using the Dead Cell Apoptosis Kit according to the manufacturer's instructions. These stained cells were observed using a fluorescence microscope (model BZ-9000; Keyence, Osaka, Japan).

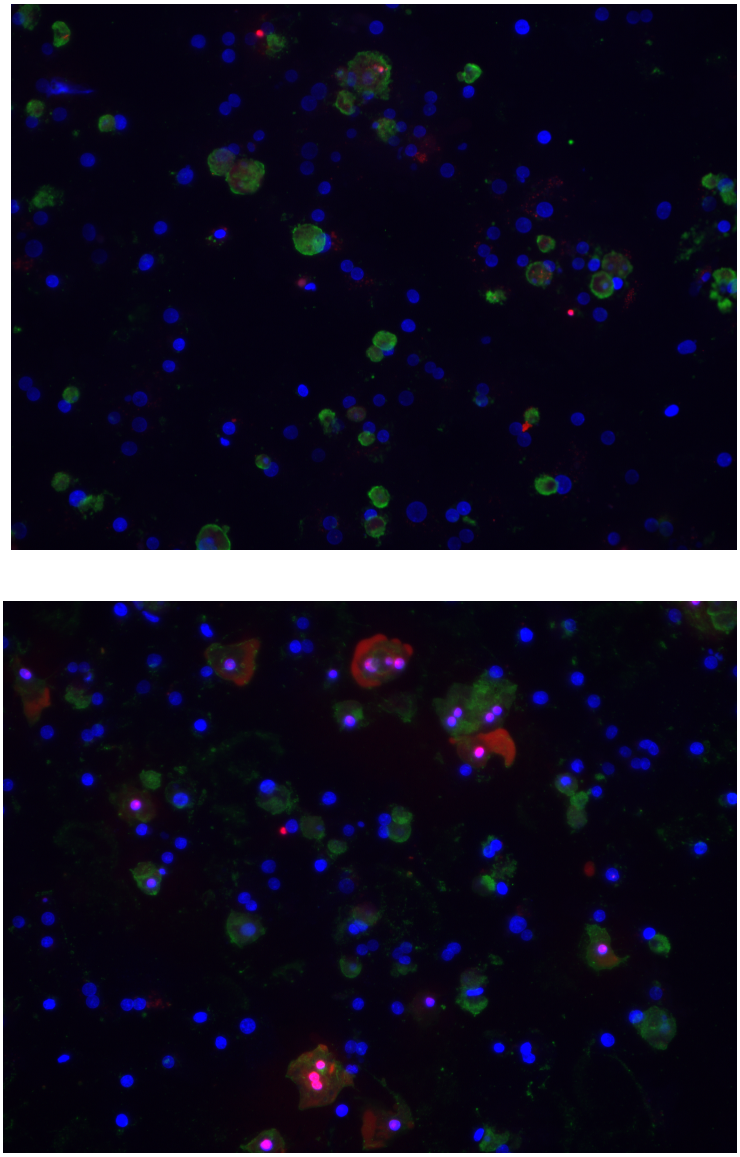

Control

GA

Supplementary Figure S1.
Annexin V/PI double staining of human primary hepatocytes. Human primary hepatocytes were treated with 4 mM of GA for 24 h and stained with Annexin V/PI . Annexin V-positive cells (green fluorescence) indicate early apoptosis. PI-positive cells (red fluorescence) indicate necrosis.

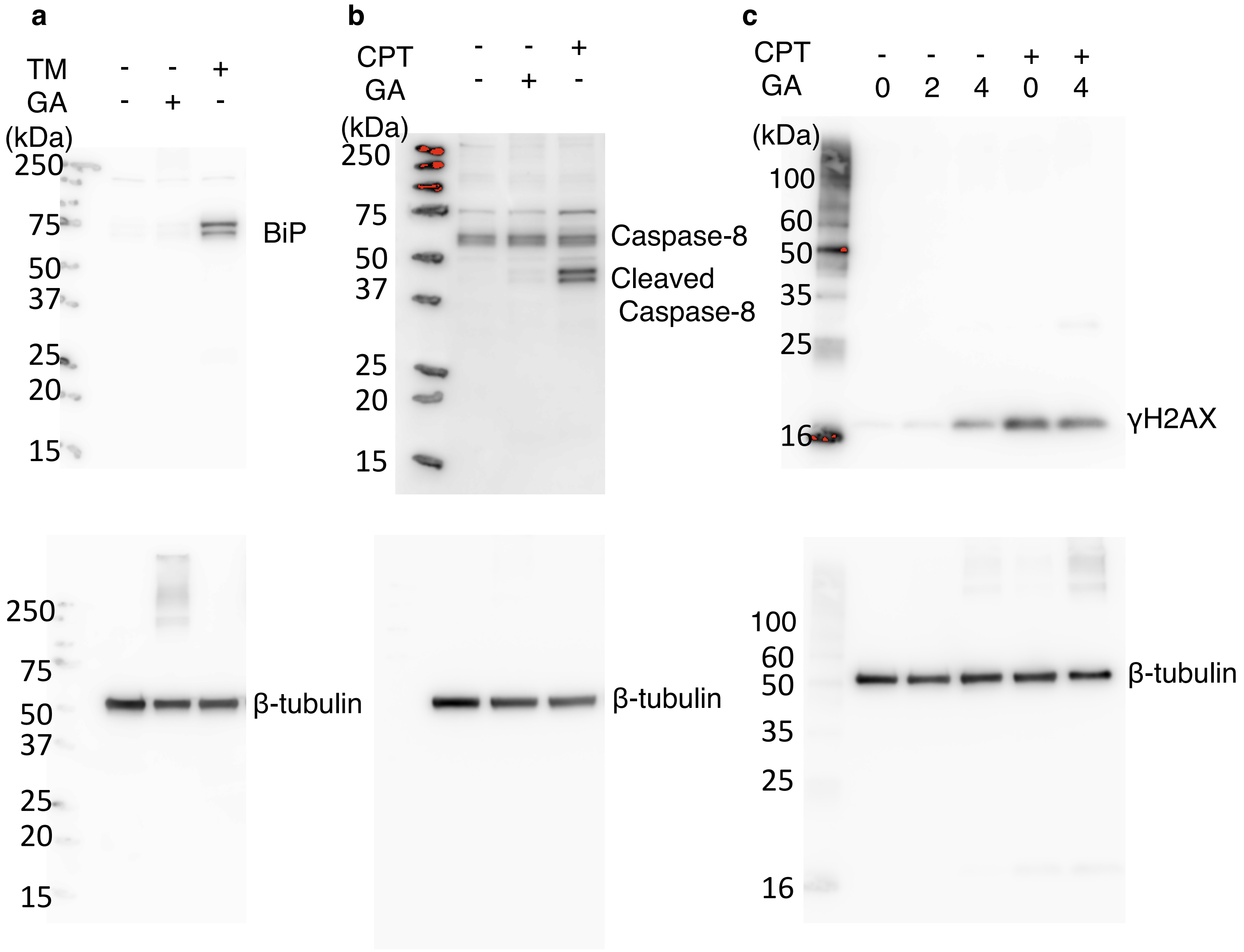

Supplementary Figure S2.
Full-length blot of Figure 2a-c.

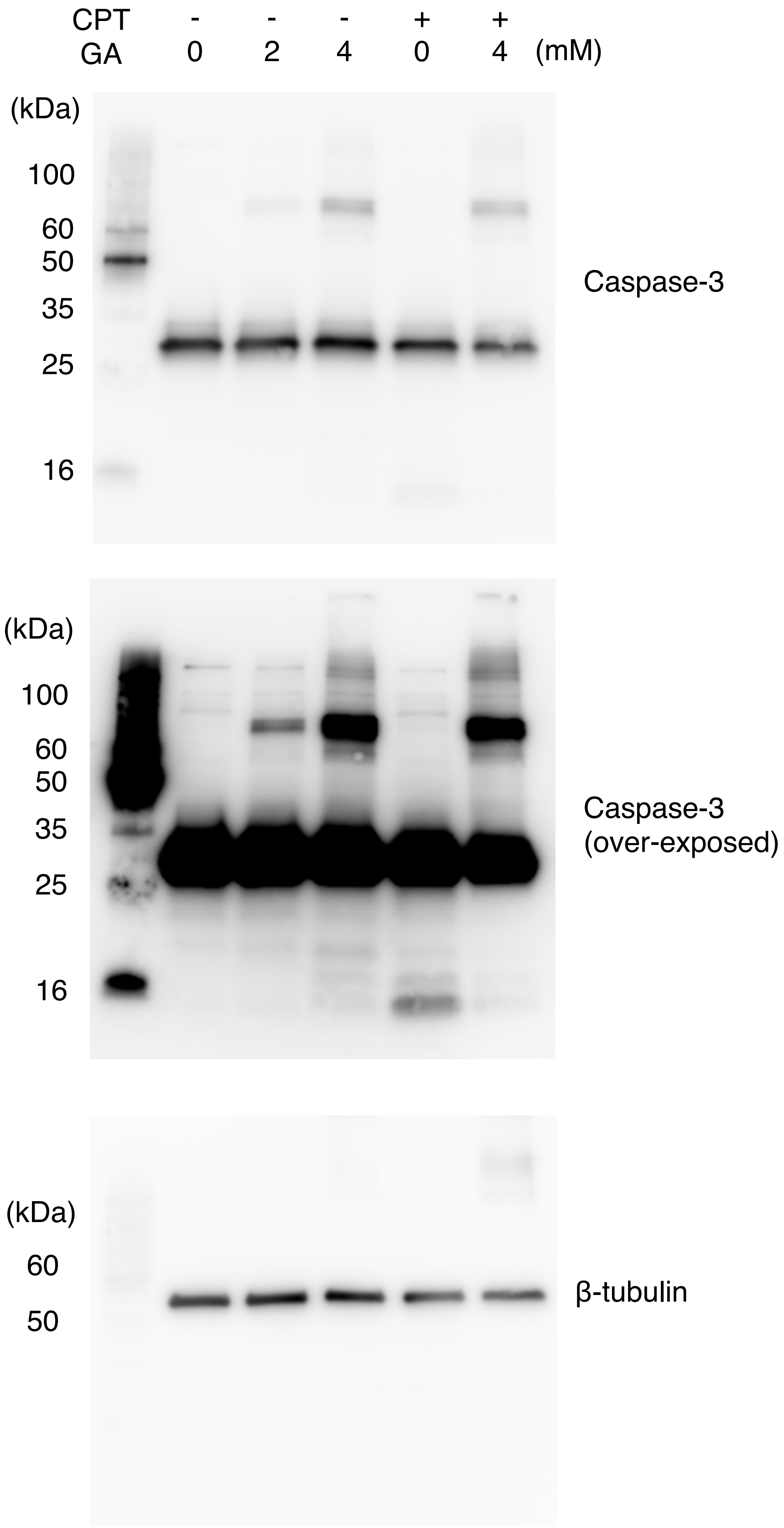

Supplementary Figure S3.
Full-length blot of Figure 4a. The middle panel of the blot was over-exposed on purpose in order to detect cleaved caspase-3.

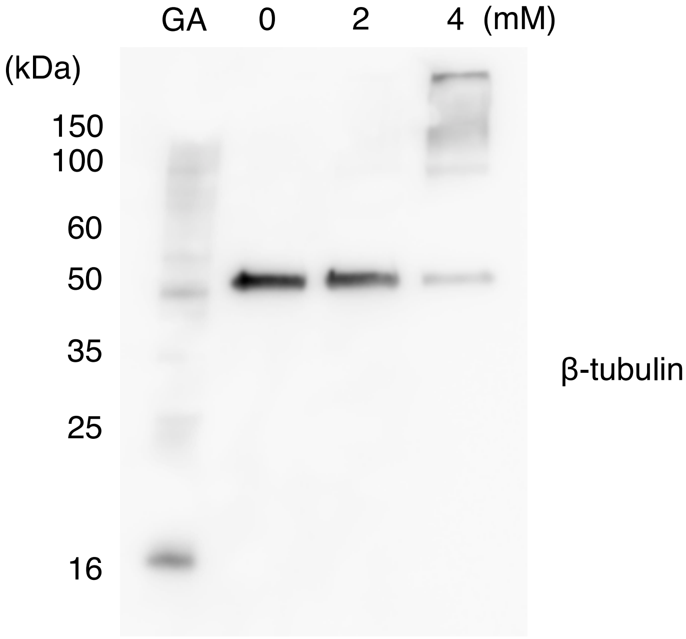

Supplementary Figure S4.
Full-length blot of β-tubulin in Figure 4b.

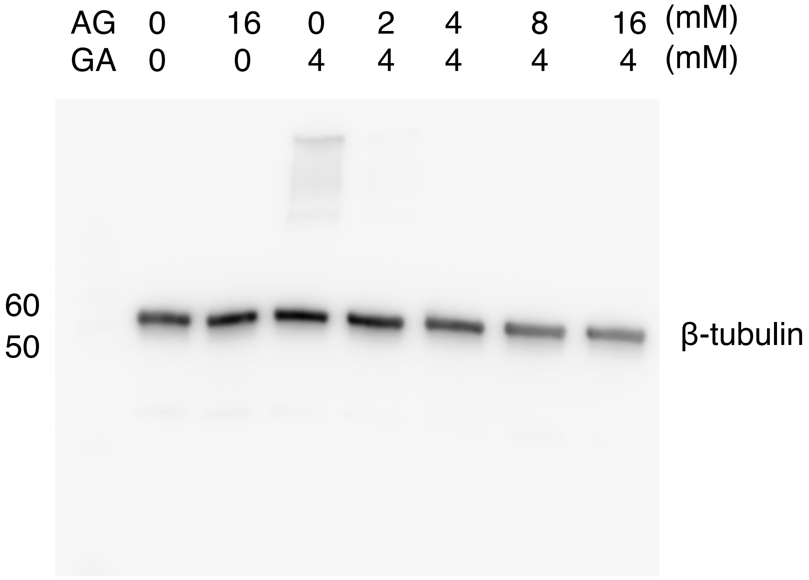

Supplementary Figure S5.
Full-length blot of β-tubulin in Figure 4c.

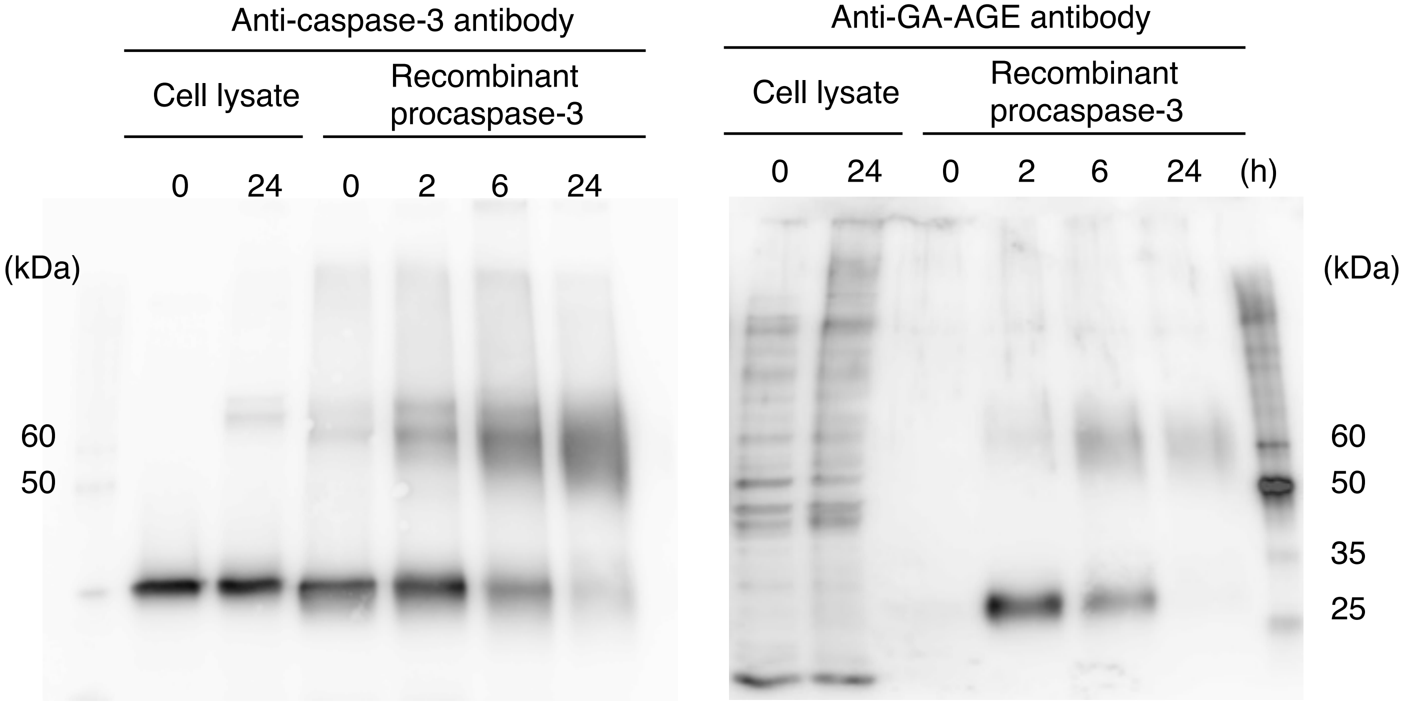

Supplementary Figure S6.
Full-length blot of Figure 4d.

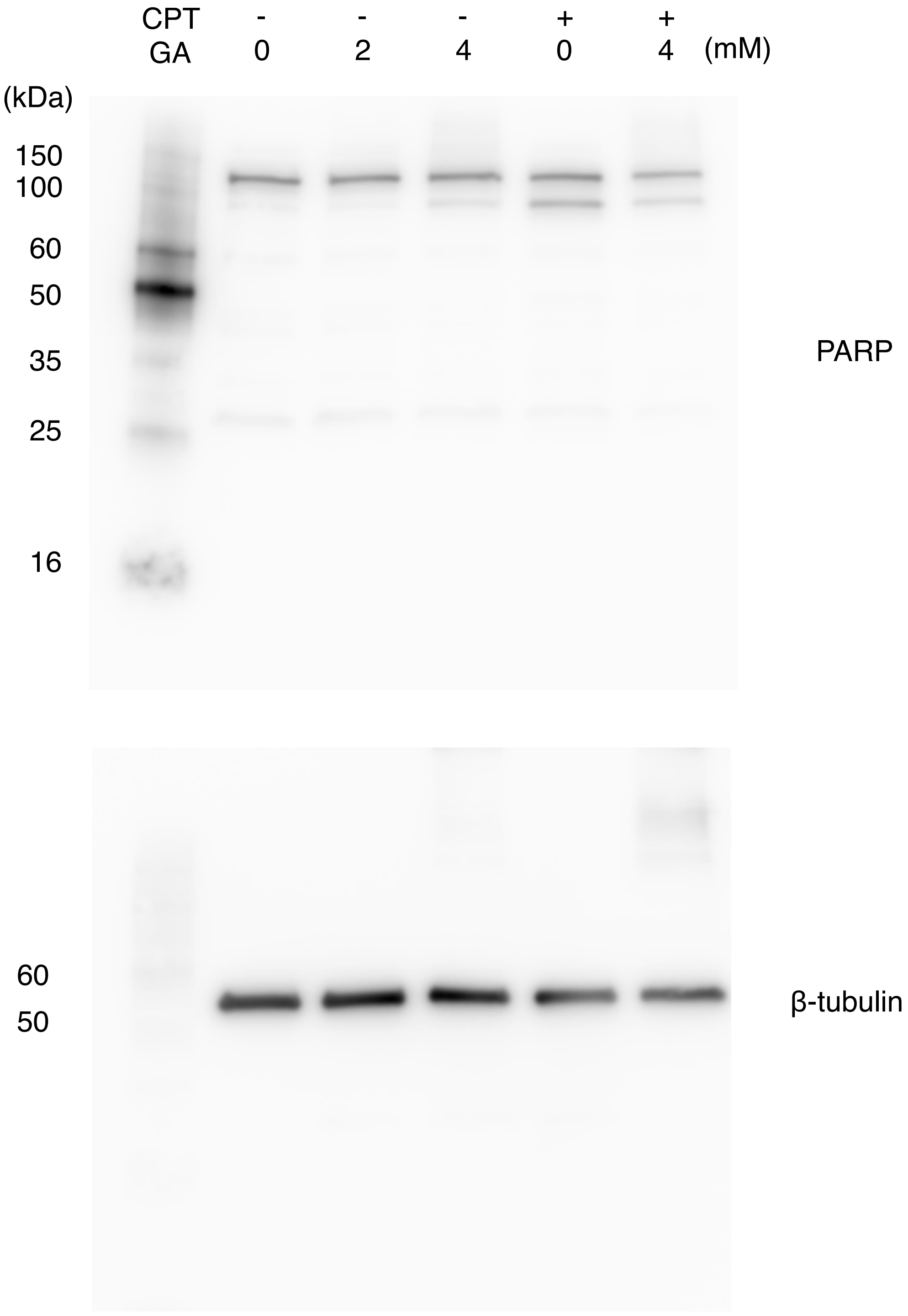

Supplementary Figure S7.
Full-length blot of Figure 5b.
